# Personal Exposure Estimates via Portable and Wireless Sensing and Reporting of Particulate Pollution

**DOI:** 10.3390/ijerph17030843

**Published:** 2020-01-29

**Authors:** Harsshit Agrawaal, Courtney Jones, J.E. Thompson

**Affiliations:** Department of Chemistry & Biochemistry, Texas Tech University, Box 41061, Lubbock, TX 79409-1061, USA; Harsshit.Agrawaal@ttu.edu (H.A.); Courtney.K.Jones@ttu.edu (C.J.)

**Keywords:** air quality, crowd-sensing, crowd-sourced sensing, environmental analysis, pollution, particulate matter, dust sensor, human exposure, Arduino, wireless networks, IoT

## Abstract

Low-cost, portable particle sensors (n = 3) were designed, constructed, and used to monitor human exposure to particle pollution at various locations and times in Lubbock, TX. The air sensors consisted of a Sharp GP2Y1010AU0F dust sensor interfaced to an Arduino Uno R3, and a FONA808 3G communications module. The Arduino Uno was used to receive the signal from calibrated dust sensors to provide a concentration (µg/m^3^) of suspended particulate matter and coordinate wireless transmission of data via the 3G cellular network. Prior to use for monitoring, dust sensors were calibrated against a reference aerosol monitor (RAM-1) operating independently. Sodium chloride particles were generated inside of a 3.6 m^3^ mixing chamber while the RAM-1 and each dust sensor recorded signals and calibration was achieved for each dust sensor independently of others by direct comparison with the RAM-1 reading. In an effort to improve the quality of the data stream, the effect of averaging replicate individual pulses of the Sharp sensor when analyzing zero air has been studied. Averaging data points exponentially reduces standard deviation for all sensors with n < 2000 averages but averaging produced diminishing returns after approx. 2000 averages. The sensors exhibited standard deviations for replicate measurements of 3–6 µg/m^3^ and corresponding 3σ detection limits of 9–18 µg/m^3^ when 2000 pulses of the dust sensor LED were averaged over an approx. 2 min data collection/transmission cycle. To demonstrate portable monitoring, concentration values from the dust sensors were sent wirelessly in real time to a *ThingSpeak* channel, while tracking the sensor’s latitude and longitude using an on-board Global Positioning System (GPS) sensor. Outdoor and indoor air quality measurements were made at different places and times while human volunteers carried sensors. The measurements indicated walking by restaurants and cooking at home increased the exposure to particulate matter. The construction of the dust sensors and data collected from this research enhance the current research by describing an open-source concept and providing initial measurements. In principle, sensors can be massively multiplexed and used to generate real-time maps of particulate matter around a given location.

## 1. Introduction

### 1.1. Summary of Problem

High concentrations of air pollution are due to fine solids, gases, or liquid aerosols locally releasing into the atmosphere or being produced at a faster rate than the environment can dilute, absorb, or dissipate the material [1]. If the rate of production is sufficiently high, substances can build up and reach a high concentration in the air that can contribute to a host of adverse health effects for humans such as cardiovascular mortality and respiratory distress [2,3,4,5,6]. Miller et al. found an increase of 10 µg/m^3^ in particulate matter mass concentration was associated with a 14% (95% confidence interval, 3%–26%) increase in nonfatal cardiovascular events and with a 32% (95% CI, 1%–73%) increase in fatal cardiovascular events [7]. Exposure to air pollution whether long or short can lead to asthma, decreased lung function, and infections of the respiratory system. Goss et al. found PM_2.5_ was associated with statistically significant declines in lung function and an increase in the odds of two or more pulmonary exacerbations in patients >6 years of age with cystic fibrosis [8]. Woodruff et al. found that each 10 µg/m^3^ increase in PM_2.5_ was associated with a near doubling of the risk of post neonatal death because of respiratory causes [9]. Hoek et al. found the risk of cardiopulmonary mortality nearly doubled for individuals who lived within 100 m of a freeway or within 50 m of a major urban road [10]. Clearly, particulate matter presents a clear and present danger to human health.

Aerosols are found everywhere including in the air over oceans, deserts, mountains, forests and ice, with the most abundant being from natural sources such as sea-spray and wind-blown soil dust [11]. For instance, dry areas of North Africa emit 800 Tgy^−1^ of soil dust each year and summertime winds can transport the Saharan dust across the Atlantic Ocean, the Caribbean, and to southern North America [12,13,14,15]. Urban areas add to the airborne particulate mass through direct emissions into the atmosphere and through emitting gases that can react in the atmosphere to form secondary aerosol. A significant body of research has gone into understanding and controlling the chemical and physical transformations that lead to secondary aerosol formation [16,17,18]. Despite the great success of air quality improvement programs implemented in Western cities over the preceding 50 years, urban environments typically have higher levels of particulate pollution compared to rural locations as cities remain global hubs of fossil fuel combustion and direct emissions [19,20,21,22,23]. An emerging threat is found in some of the world’s poorest cities as the World Health Organization (W.H.O.) has recently reported 98% of cities in low and middle income countries with more than 100,000 inhabitants do not meet WHO air quality guidelines [24]. Aerosols are also present indoors and homemakers in economically disadvantaged environments can often be exposed to very high levels of particulate matter when using home cook-stoves to prepare meals [25,26]. Monitoring and controlling human exposure to PM_2.5_ remains a crucial scientific challenge and this focus requires the continued development and application of portable and low–cost sensing platforms for widespread application.

### 1.2. Current Work in the Field

Historically, PM_2.5_ concentrations have been measured using either inertial impactors, filter based sampling, or sophisticated laboratory-based measurement devices [27,28,29,30,31]. However, such approaches are either too expensive, too labor intense, too slow, too limited in scope, or all of the above to address the needs of the problem. A new paradigm is needed in which many end users can access accurate, real-time data at very low cost to society [32,33].

Several brands (Shinyei, Sharp, Nova, Plantower, Wuhan, Alphasense, Air Beam, etc.) of low-cost electronic sensors that use light scattering can be used to monitor particulate matter disbursed in air. These sensors come at different price points and sizes but are designed to use low power and can operate at high measurement frequency. The common limitation in the sensors is the accuracy and the reproducibility of the data, and very limited ability to measure particles below 200–300 nm in diameter due to low scattering cross section. In addition, low-cost sensors often have limited linear dynamic range, and often plateau or ‘max out’ at a few hundred microgram per cubic meter concentration. This feature of devices may underestimate the impact of high exposure events.

Currently, one of the most successful integrated approaches to particulate matter monitoring is called Airbeam (habitatmap.org). In this project, a palm-sized device costing $250 measures particulate pollution that a user is exposed to by a light scattering method and transmits data to a co-located Android device through a Bluetooth connection. The wireless connection on the secondary device and an Android OS App is used to ‘AirCast’ collected data to a central data server and maps are created by combining various end users’ data streams. The integrated approach demonstrates the potential of the crowd-sourced architecture. 

The excitement generated by the initial emergence of low-cost sensing platforms such as ‘Airbeam’ has led the Environmental Protection agency (EPA) of a major North American country to begin evaluating the performance of low-cost sensors co-located with sensors their government considers “reference” and “equivalent” methods (see https://www.epa.gov/air-sensor-toolbox). In an exclusive project, in 2019 this EPA began a study aimed to tackle questions about long-term performance of low-cost air sensors, an area that is rarely (if ever) explored in academic settings. The exclusive group of EPA funded staff are currently evaluating six different models of low-cost air sensors, placed at seven diverse locations throughout states within North America. The locations have diverse climates and air quality conditions, assuring that the project produces a dataset that investigators may use to assess how weather conditions affect sensor signals and long-term performance. 

In Gunawan et al., a suite of portable sensors (PM_2.5_, PM_10_, CO, O_3_) were used in Malaysia to derive the local air pollution index (API) at an end user’s location [34]. The API was reported to the user in nearly real-time thru an LCD display. For carbon monoxide a model MQ-9 sensor was employed, while ozone was monitored with the MQ-131 gas sensor. The Sharp dust sensor was used to measure PM_10_ while a Shinyei PPD42NS was used to derive PM_1-2.5_. The set-up was controlled by the Arduino microcontroller, and the authors reported a cellular modem and Global Positioning System (GPS) card could be added if desired. However, wireless communication was not a focus of the work. Unfortunately, the authors were unable to perform proper calibration of each sensor and had to rely on suggested calibration equations from the manufacturer. In addition, laboratory validation measurements were not completed and data quality unassessed. 

In Reilly et al., an affordable monitoring device costing ≈$280 was constructed [35]. The device can send and collect data to a real-time mapping program wirelessly using the Global System for Mobile Communications (GSM) to a server, or as a text message to people nearby. The device consisted of the Redboard Arduino clone, a GSM board, a carbon monoxide sensor, an ozone sensor, and Sharp dust sensor for particulate matter (PM), a fan to promote airflow, and instrument case. While the authors report successful implementation of their device, environmental sensors were not calibrated nor rigorously tested in a laboratory. Instead, the authors used the recommended calibration equations from the product manufacturers to convert electrical signal to environmental measurement. Suspiciously high and constant PM levels were reported (approx. 150 µg/m^3^) and ozone levels did not exhibit the characteristic diurnal pattern that is observed nearly everywhere in the troposphere. Consequently, despite the approach being successfully implemented, the quality of environmental sensor results is questionable.

In Zamora et al., Plantower AMS A003 sensors were tested in the laboratory to measure particulate matter of various composition in a series of well-defined and controlled experiments [36]. The sensors were precise; however, when the sensor results were compared with a reference method, accuracy varied as a function of aerosol composition and humidity. The sensors were highly precise with a R^2^ values greater than 0.86 for all sources. However, the accuracy had a large range from 13% to greater than 90% compared with reference instruments, depending upon aerosol type. The sensors were more accurate when the particulate matter was spherical and smaller than 1 µm in size. The sensors’ accuracy was greatly affected by the humidity. The work of Zamora et al. contributes significantly to the field because it draws attention to the limits of single angle nephelometry to estimate PM mass concentration vis-à-vis the aerosol composition and shape. While the sensors were able to provide usable data when in motion or in high or low temperatures, the focus of this manuscript was refreshingly on data quality. Results suggest that portable sensors should be calibrated versus an accepted reference method using authentic aerosol as a sample. 

Liu et al. takes this approach by examining performance of Nova particulate matter sensors (SDS011) [37]. The sensors were tested on ambient samples in Oslo, using a co-located, official air quality monitor as a reference method. All of the sensors had results that were similar including inter-sensory correlations with R^2^ values greater than 0.97. Again, high humidity greatly affected the sensors. There was a linear relationship between the sensors and the reference monitor. However, the R^2^ varied over the range of 0.55 to 0.71. When a data correction using relative humidity and temperature was used, the R^2^ value for each sensor increased from 0.71 to 0.80, 0.68 to 0.79 and 0.55 to 0.76, respectively. These sensors were also limited by the environmental conditions and can have high error if used outside of the manufacturer specifications. This work draws attention to the need to calibrate versus an accepted gravimetric reference method with authentic aerosol and the need to correct measurements (or at least flag them) for conditions of high humidity.

Liu et al. have compared Shinyei, Sharp, and Oneair optical sensors in laboratory experiments with polydisperse particles of a variety of compositions, concentration, and mean size [38]. These authors found the mass concentration normalized response of all optical sensors clearly changed with mean particle size even when considering the narrow range of very small particles between 70–95 nm. The composition of particles was even more crucial—particles of methylene blue, sodium fluorescein, and sodium chloride all produced sensitivities differing by at least 50% (with NaCl being lowest). These results clearly demonstrate that choice of calibrant aerosol is crucial for obtaining accurate sensor mass calibrations. The current manuscript is not exempt from such considerations or potential errors. 

### 1.3. Contribution of the Current Work

The current study aims to build upon previous work from our laboratory in which a field-portable device for logging PM_2.5_ mass concentration data was developed [39]. The previous device also used the Arduino, and a Sharp sensor with nephelometric detection, but logged all collected data to an SD card. This prevented real-time feedback to the user and complicates integrating/multiplexing many sensors together to form a network of streaming data. This limitation has recently been addressed thru our laboratory’s development of an open-source data acquisition platform we have called *logIT* that wirelessly transmits data over the 3G cellular network [40]. Source code to implement *logIT* is available online for the user community [40]. The *logIT* platform has allowed multiplexing multiple sensors (here n = 3) to demonstrate multi-user simultaneous measurements of PM exposure throughout a mid-sized city. In addition, we build upon the previous literature work using the Sharp GP2Y1010AU dust sensors [34,35] by performing laboratory measurements to characterize and constrain device performance. This was not reported in [34,35] and results presented within this manuscript will allow the community to make more informed decisions regarding the performance characteristics of Sharp GP2Y1010AU dust sensors. The current manuscript also serves as a companion article to [38], as the current work highlights signal to noise considerations and intercomparisons of individual Sharp dust sensors themselves, while Liu et al. [38] provide much more through insight into calibration of low-cost sensors against a gravimetric equivalent method and the crucial effect of calibrant composition and size. 

## 2. Methodology 

**Figure 1:**
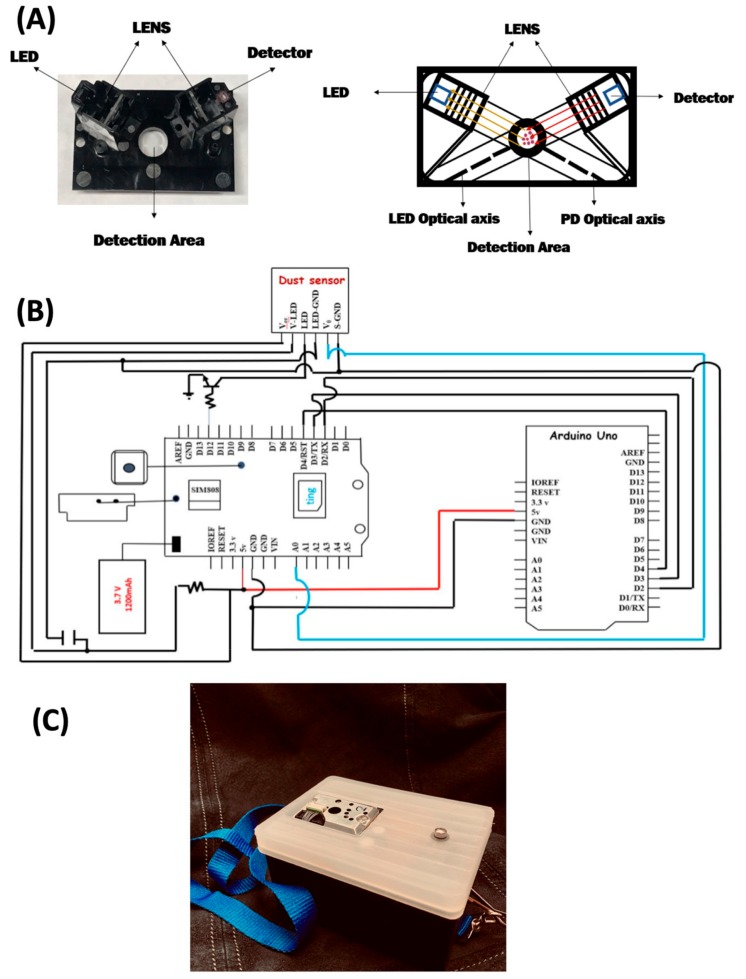
(**A**) Photo and drawing of Sharp GP2Y1010AU0F dust sensor. (**B**) Wiring schematic for the dust sensor and FONA module to the Arduino (C) Photograph of the portable project box containing the dust sensor, FONA module and Arduino. The project box is 12 cm × 9 cm × 6.5 cm and has a mass of 250 g.

Three Arduino Uno R3 ($14.99 USD each), FONA808 modules (Adafruit, $49.95 USD each) and dust sensors (GP2Y1010AU0F $15.23 USD each) were purchased and used as received without modification. The dust sensor is a nephelometric sensor illuminating sample with a near infrared LED with a wavelength of 860 nm and collecting light at 120 degrees from incident illumination as depicted in Figure 1A. The dust sensors were affixed to circuit boards with cyanoacrylate and wired into Arduino ‘stackable shields’ in house by laboratory staff. Stackable shields plug into the Arduino board directly by using header pins for both electrical and mechanical connection. The wiring connections used are illustrated in the circuit diagram of Figure 1B. The FONA808, Arduino Uno, and dust sensors were mounted into plastic project boxes as depicted in Figure 1C. For user comfort, a lanyard was attached to each box. The Arduino Uno was used to control FONA808 modules and dust sensors. FONA808 communication modules were used for reporting the particulate matter concentration to a Thingspeak.com channel. Technical details of this process [40]. The data was collected in real time and logged to Thingspeak.com until downloaded for analysis. All the calibrations were performed with wireless communication with 9V power supply powering the dust sensor modules. 

The Sharp dust sensors produce an analog voltage as output signal. To convert the analog signal to a meaningful PM concentration, the dust sensor must be calibrated against references before making meaningful measurements. A commercial device, the reference aerosol monitor (RAM-1) from Monitoring Instruments for the Environment (MIE, Inc., Billerica, MA, USA) was chosen as a reference method to calibrate each sensor. The RAM-1 actively samples aerosol using a pump and estimates concentration using the light scattering principle. The RAM-1 analysis is not an EPA equivalent method for PM_2.5_. We have performed four trials in which the RAM-1 sensor was used to compare the indicated concentration of a sodium chloride test aerosol within a chamber with gravimetric determination of aerosol mass concentration. A 37 mm quartz filter was used to collect a sodium chloride test aerosol at 15 SLPM using a mass flow controller and vacuum pump. Gravimetric measurements of (average) concentration over the sampling period could be obtained and compared directly with the RAM-1 values. For these 4 trials we find that the RAM reading is on average 107% (Std Dev. = 67%) of the gravimetric result with n = 4.

For calibration, all Sharp dust sensors were placed inside a 3.6 m^3^ volume chamber lined with a fluoropolymer (FEP). Each dust sensor was individually calibrated in the 3.6 m^3^ chamber. A single jet atomizer (TSI 9302) generated sodium chloride particles into the chamber with an atomizer pressure of 20 psi. The flow of particles entering the chamber was 5.7 L min^−1^. The jet generated polydisperse sodium chloride particles into the chamber. After approximately 2 min, sufficient aerosol was produced and the atomizer jet was shut off for the remainder of the experiment. The relative humidity inside the chamber under these conditions was measured to be < 20%, indicating presence of a dry aerosol. Experimental runs at high relative humidity were not attempted, and performance under these conditions not explored in this study. The calibration data was then collected for 4–5 h as the initially high particle concentration dissipated due to impaction on the chamber walls. For each point in time, the individual sensors were compared to the indicated concentration of the reference aerosol instrument (RAM) placed inside the chamber. Each dust sensor was calibrated individually, and the process was repeated for each sensor. All of the data from the dust sensors were uploaded to the ThingSpeak channel in real time using the FONA808 module.

## 3. Results and Discussion

### 3.1. Optimizing Delay Time

**Figure 2:**
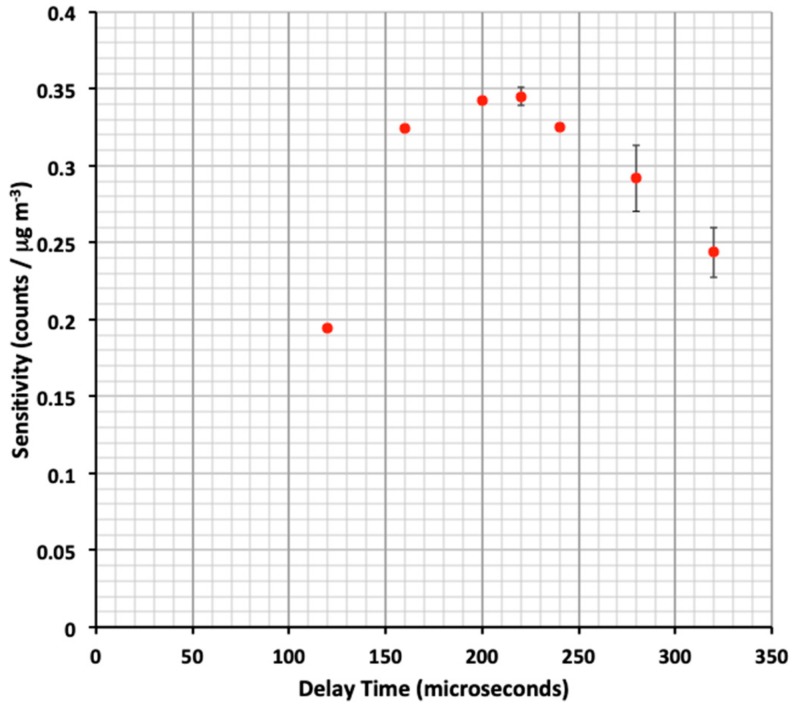
Plot of Sharp GP2Y1010AU0 dust sensor sensitivity vs. delay time prior to signal acquisition. The sensitivity is the slope of the best-fit calibration line when signal was plotted vs. PM mass concentration (µg/m^3^). The optimal delay of 220 microseconds was employed for subsequent measurements. Error bars represent ±1σ of replicate trials.

The Sharp dust sensors operate by turning on a near-IR LED, waiting a user-specified delay period, and then sampling the scattered light signal prior to cycling the LED off again. The entire measurement cycle can be completed in under a millisecond and this measurement cycle can repeat itself many times. Indeed, signals can be averaged to improve limits of detection (see section below). The LED is turned on by a HI/LO (5 - 0 Volt) transition at a digital pin on the microcontroller, and a delay prior to signal acquisition is then initiated. While Sharp recommends a delay time of 280 µsec prior to collection of data in product literature, we have systematically studied the effect of delay time on sensitivity (slope of calibration line). Figure 2 reports the results of this study. As observed, measurements suggest that a 220 microsecond delay after the LED trigger allowed optimal sensitivity to be achieved for the individual sensors tested in this study. The cause of the shift in optimum delay time from the manufacturer’s recommendation is not known. However, differences in stray capacitance between our apparatus and the manufacturer’s test bed may be the cause. The result suggests end users may wish to optimize delay time for their own application. Regardless of the cause, the delay time has been optimized to provide maximum sensitivity for this study.

### 3.2. Sensor Calibration Results

**Figure 3:**
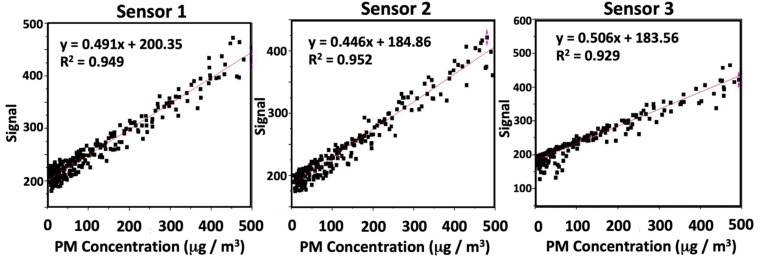
Calibration plots for sensors 1–3. Observed signal from the Sharp GP2Y1010AU0 dust sensors vs. PM concentration indicated by the RAM reference method for laboratory generated polydisperse NaCl aerosol. Observed signal is the digital count assigned by the Arduino board during analog-to-digital conversion. Differences were found in slope and intercept between the sensors tested, and R^2^ > 0.92 observed. Relative humidity within the 3.6 m^3^ chamber during calibration was < 20%.

Figure 3 illustrates plots of dust sensor signals vs. indicated PM mass concentration for the three dust sensors used in this study. In these experiments, signal from a commercial PM mass concentration monitor (RAM) was used as the reference/accepted concentration. Our analysis is limited in we assume no error, uncertainty or imprecision is present in the RAM data stream. For all sensors, there was a linear and positive correlation (R^2^ > 0.92) observed between the concentration (µg m^−3^) and dust sensor signal to 500 µg m^−3^. A linear-least squares best-fit line was added to each dataset and used to determine the slope and intercept for each sensor. The slopes were 0.491, 0.446, and 0.506 digital counts per µg m^−3^ for sensors 1, 2 and 3. Since the Arduino analog acquisition is a 10 bit device operating over a full span of 1024 steps from 0 to 5 V, a digital step corresponds to 4.88 mV/count. Therefore, our sensors were observed to produce between 0.217 and 0.247 V per 100 µg/m^3^ or roughly half of the signal specified by the product data sheet for the Sharp dust sensor which specifies 0.5 V signal per 100 µg/m^3^ of PM mass concentration [41]. The exact cause of this discrepancy in sensitivity remains unclear. However, we note that Liu et al. [38] previously observed sodium chloride aerosol produced a much lower signal compared to other compositions. Varying the size distribution or particle shape of the test aerosol will lead to differences in differential scattering cross-section at 120 deg. and consequently, the observed slope of calibration lines. Emerging research on low-cost dust sensors [36,38] has provided clear evidence that the light scattering method can be subject to large biases when projecting mass concentrations when calibrations were conducted against aerosol with different properties (refractive index, shape, size, composition) than the ambient type encountered at a particular location or for a particular application. As such, this could be responsible for the low sensor responses we report. 

### 3.3. Effect of Signal Averaging

**Figure 4:**
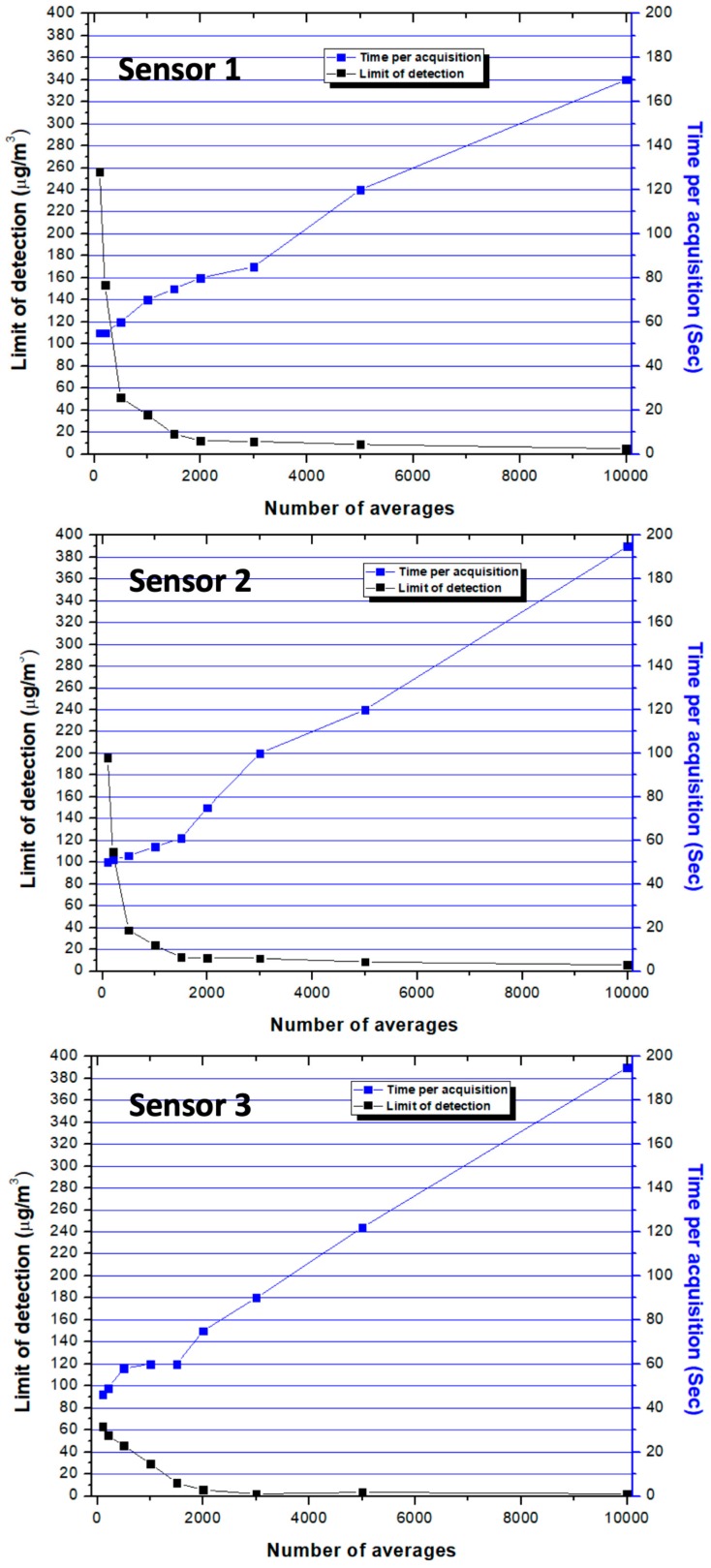
The effect of number of LED pulses averaged (N) on the limit of detection (black squares) for sensors 1–3. The second y-axis (blue) presents the seconds required to acquire and report data for the specified number of averages.

Since the data acquisition cycle for a single pulse of the LED is very short (ms time), averaging many pulses from the LED is an attractive approach to enhancing detection limits for the Sharp dust sensors. Code can easily be added to the Arduino sketch to accomplish signal averaging in the onboard memory of the microprocessor on the fly. Only the averaged result is then reported in the data stream. A study was carried out in which an air blank was analyzed, and the number of LED pulses averaged together were 100, 200, 500, 1000, 1500, 2000, 5000, and 10,000 individual pulses. Data points were reported for each average and the standard deviation (σ) of replicate averages computed. Then, the limit of detection (L.O.D.) for each sensor was computed by using the 3σ standard. As observed in the black trace of Figure 4, as the number of LED pulses averaged increased to roughly n = 2000, the measurement L.O.D. decreased an order of magnitude for all the sensors tested. Interestingly, averaging approx. 2000 pulses only marginally affects the total time required to report a single data point (shown in blue data series) because the wireless communication protocol alone requires nearly a minute on average to complete its reporting cycle. Figure 4 also demonstrates that if additional LED pulses > 2000 were averaged together, there were only marginal additional gains in limit of detection achieved. However, the time required for data acquisition when n > 2000 began to linearly increase with the number of averages since wireless communication was no longer the rate limiting step under these conditions. Consequently, 2000 averages were used in subsequent monitoring activities. Under these conditions, the standard deviation and limit of detection for the three sensors were between σ = 3–6 µg/m^3^ and L.O.D. = 9–18 µg/m^3^, respectively. These results indicate that Sharp dust sensors and microprocessor mediated signal averaging can be used to track PM pollution within environments where substantial particle pollution is expected. This could include routine workplace monitoring or personal exposure monitoring for citizens living in urban centers where levels regularly exceed 25–30 µg/m^3^. However, it should be noted that the method we describe herein is not sensitive enough for monitoring in all environments (see below). Further improvements in precision, limits of detection, and sensor accuracy are still required to bring ambient portable sensing to its full potential.

### 3.4. Precision of Dust Sensors

**Figure 5:**
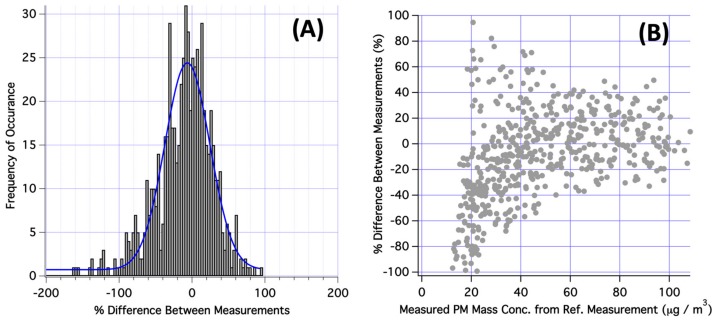
(**A**) Histogram of percent difference between individual Sharp GP2Y1010AU0 dust sensor measurements and the reference method measurements (RAM-1) for test sodium chloride aerosol within a chamber. A Gaussian fit to the data indicated σ = 30.8 for the entire comparison dataset. (**B**) Plot of percent difference between Sharp dust sensor and the reference measurement plotted vs. the indicated PM mass concentration for the reference method.

Figure 5A reports a histogram of observed percent difference between individual Sharp dust sensor measurements and the reference method (RAM) measurements for test sodium chloride aerosol within a laboratory chamber. Figure 5 includes data points from all 3 Sharp dust sensors. Here, the percent difference was computed as (Sharp sensor measurement–reference measurement)/reference measurement and the result expressed as parts-per-hundred relative difference. As observed in Figure 5A, results were observed to follow a normal distribution. A Gaussian fit to the data indicated a standard deviation of σ = 30.8 for the entire dataset. This result suggests, considering all data, Sharp sensors report concentrations within 30.8% of the reference value about 68% of the time. The standard deviation also allows end users to define confidence intervals for their data points. In Figure 5B, a plot of percent difference between Sharp dust sensor and the reference measurement is plotted vs. the indicated PM mass concentration for the reference method. This plot indicates that the largest relative discrepancies between dust sensor indicated mass and reference monitor mass occurred when PM mass concentration was < 40 µg m^−3^. Above this mass loading, relative difference between the dust sensor and reference monitor was very frequently < 20%. Considering only data collected when PM > 40 µg m^−3^, the average percent difference between a Sharp dust sensor and reference measurement for single point comparison was 5.8% and the median absolute percent difference was 15.2%. This result indicates that unmodified Sharp dust sensors offer best precision when PM mass loadings are relatively high (e.g., PM > 40 µg m^−3^). 

### 3.5. Monitoring Experiments

**Figure 6:**
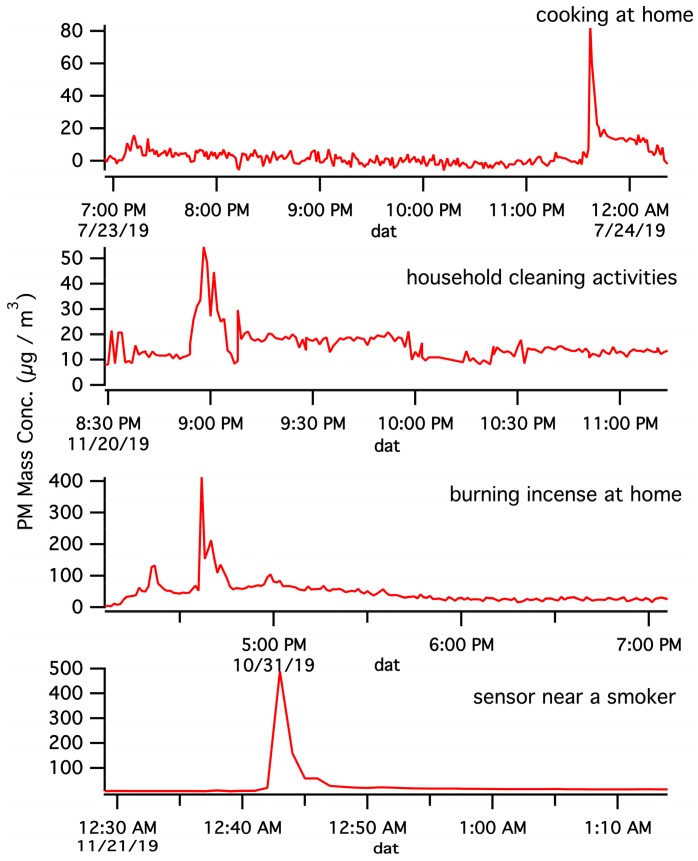
Sample monitoring results for end users during the trial implementation. Real-time personal exposure monitoring allows improved understanding of the times, locations, and extent of high exposure events. The sensors were in the same room adjacent to the end user during the events.

The ultimate goal of this work is to improve understanding of human exposure to PM pollution through creating and implementation of a network of multiplexed, portable PM sensors. In this manuscript, we report a limited scope proof-of-concept application by having laboratory personnel carry the three dust sensors around Lubbock, TX. The staff engaged in normal life activities during sampling in an effort to demonstrate that data from multiple sensors can simultaneously be streamed to a Thingspeak channel, while the end user can access information about his/her own exposure in nearly real-time via a public Thingspeak web link. Figure 6 reports example time-series monitoring data that end users encountered during the effort. As observed, spikes in PM mass loadings were encountered by the users during random life events such as walking near an individual smoking cigarettes, cleaning an apartment space, burning incense at home, or cooking. It is often difficult to accurately parameterize exposure during such casual life experiences in models of human exposure to PM pollution, and portable PM sensors have great promise to improve quantification of PM for such circumstances. In an effort to improve understanding of human exposure in different environments we have performed measurements of PM mass concentration at a variety of locations and report a summary of these measurements in Table 1 of this manuscript. While a wide range of PM levels were encountered across locations (and even at single locations through time), the reported levels begin to improve understanding of typical levels of human exposure in various environments. For instance, results have suggested that human exposure to PM is often quite high near any cooking operation, or at some restaurants. The use of low-cost, portable sensors to uncover and document such knowledge can be used to develop exposure control strategies for workers in these environments. 

**Figure 7:**
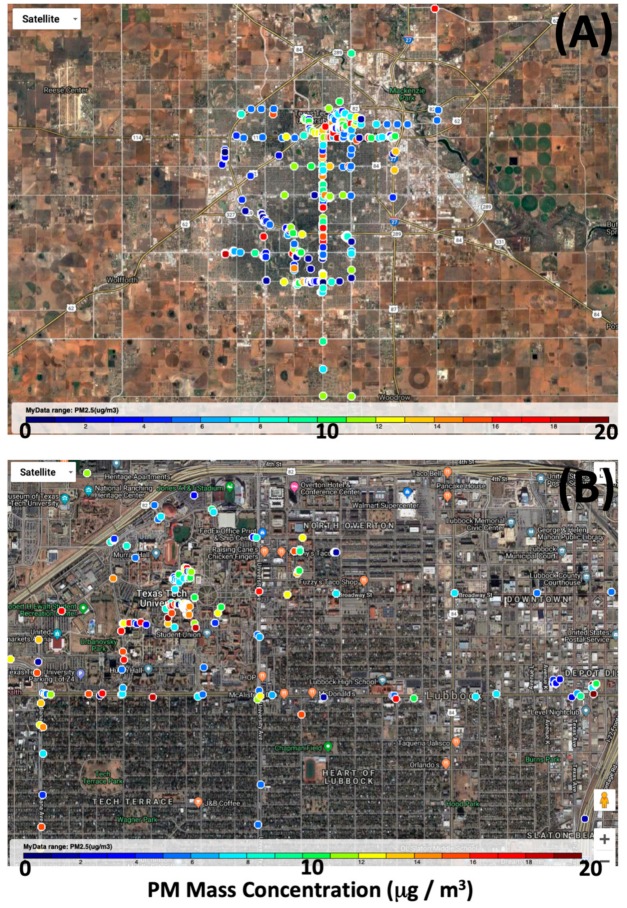
Maps of Lubbock, TX with user sampled PM single-point data superimposed. Mobile sensors transmitted GPS coordinates and dust sensor PM measurements to a webserver, and maps were created from data. Map in (**A**) is approx. 40 km wide by 28 km high. Map in (**B**) is zoomed to illustrate specific locations sampled by users during their experiences. Data point colors describe an individual observation PM mass concentration in µg m^−3^ and are not meant to represent trends or averages.

Another goal of future research is to begin documenting the temporal and spatial behavior of aerosol levels at specific locations. Figure 7 presents a map of PM levels encountered by end users as they carried the portable sensors with them through their daily routines. Since the platform developed transmits GPS coordinates along with PM levels, data from multiple sensors can be combined and plotted. Such plots can ultimately be used to better understand the spatial dynamics of particle pollution within a city, and comprehensive datasets can better educate the public in regard to differences in pollution exposure between regions of a city or neighborhoods. However, the results presented in this manuscript do not represent such an achievement. Understanding temporal and spatial averages for PM concentrations at specific locations is a complex task that can only be accomplished through many more measurements than what we report within this paper.

## 4. Conclusions

Low-cost dust sensor modules (n = 3) were constructed using an Arduino Uno R3, a FONA808 module and a Sharp dust sensor. The modules employed the *LogIt* platform our laboratory has previously developed to wirelessly transmit GPS and particle mass concentration data to a web server [40]. The dust sensor was calibrated in the lab against a commercial optical PM mass concentration monitor and subsequently used to monitor the air quality in Lubbock, TX during daily activities, such as walking, cooking, sleeping, sweeping, traveling or working. The concentration of particulate matter was collected in real time with GPS coordinates. The optimal limits of detection for the unmodified Sharp dust sensors tested ranged from 8 µg m^−3^–17 µg m^−3^. The particulate matter concentration remained relatively low, and constant during outside measurements, but data had moderate spikes inside dwellings during certain activities. High concentrations were often observed near restaurants. Multiplexing the sensors (using many simultaneously) can better constrain human exposure to PM, and subsequently better determine health effects of particle pollution.

In the future, our sensors should be calibrated and challenged against additional aerosol compositions and size distributions to improve performance and reliability. Because particle size distribution and composition may affect sensor response we advocate for direct calibration of the Sharp sensors alongside real-time EPA federal equivalent methods such as the TEOM or beta-attenuation monitor using authentic aerosol as the sample. Such direct comparison/calibration on authentic aerosol typical of a specific location will provide best performance. Additionally, the state of the science can be advanced by developing in-field calibration protocols for the low-cost sensors to routinely assess and check sensor calibrations.

## Figures and Tables

**Table 1:** Summary of results for locations tested.

		Range of Concentration (µg/m^3^)		Statistical Indices (µg/m^3^)	
Place/Activity		Maximum	Minimum	Mean Conc.	Median Conc.
Driving		159	Below L.O.D.	15	Below L.O.D.
Living Space	Location 1	84	Below L.O.D.	Below L.O.D.	Below L.O.D.
	Location 2	186	Below L.O.D.	Below L.O.D.	Below L.O.D.
	Location 3	113	Below L.O.D.	27	18
Parking Lot	Location 1	28	Below L.O.D.	Below L.O.D.	Below L.O.D.
	Location 2	48	29	34	33
	Location 3	25	Below L.O.D.	18	19
	Location 4	16	Below L.O.D.	Below L.O.D.	Below L.O.D.
Church	Location 1	Below L.O.D.	Below L.O.D.	Below L.O.D.	Below L.O.D.
Room Renovation	Location 1	85	14	28	25
Workshop	Location 1	70	Below L.O.D.	Below L.O.D.	Below L.O.D.
Restaurant	Location 1	346	20	46	39
	Location 2	44	Below L.O.D.	24	24
	Location 3	24	Below L.O.D.	Below L.O.D.	Below L.O.D.
	Location 4	81	Below L.O.D.	46	47
Grocery Stores	Location 1	25	Below L.O.D.	Below L.O.D.	Below L.O.D.
	Location 2	22	Below L.O.D.	17	17
	Location 3	41	Below L.O.D.	26	26
	Location 4	Below L.O.D.	Below L.O.D.	Below L.O.D.	Below L.O.D.
University building	inside	39	Below L.O.D.	Below L.O.D.	Below L.O.D.
	outside	18	Below L.O.D.	Below L.O.D.	Below L.O.D.
Coffee shop	Location 1	25	Below L.O.D.	16	16
	Location 2	17	Below L.O.D.	Below L.O.D.	Below L.O.D.
	Location 3	Below L.O.D.	Below L.O.D.	Below L.O.D.	Below L.O.D.

**Note:**Table 1 data is not representative of exposure in all specified microenvironments. Human exposure to PM is highly circumstantial based upon an individual’s specific experience.
